# An experimental study of the sensorized strain wave gear RT1-T and its capabilities for torque control in robotic joints

**DOI:** 10.3389/frobt.2024.1416360

**Published:** 2024-08-09

**Authors:** Robert Schuller, Jens Reinecke, Henry Maurenbrecher, Christian Ott, Alin Albu-Schaeffer, Bastian Deutschmann, Fred Buettner, Jens Heim, Frank Benkert, Stefan Glueck

**Affiliations:** ^1^ Institute of Robotics and Mechatronics, German Aerospace Center (DLR), Wessling, Germany; ^2^ Automation and Control Institute, Faculty of Electrical Engineering and Information Technology, Wien, Austria; ^3^ Schaeffler Technologies AG & Co. KG, Schweinfurt, Germany

**Keywords:** robotic joint, torque control, joint torque sensing, sensorized strain wave gear, collaborative robot, experimental study

## Abstract

The idea of sensorizing a strain wave gear to measure the transmitted torque has been reported since the 1980s. The strain in the elastic flex spline is typically measured by strain gages attached to it. The resulting voltages relate to the transmitted torque in the gear. However, periodic inaccuracies in the measured torque signal (sensing ripple), resulting from positioning inaccuracies of strain gages on the flex spline, prevented this technology from being used outside a lab environment. Regardless of these difficulties, measuring the torque directly in the strain wave gear would bring many advantages, especially in robotic applications, where design space is highly limited. Traditionally, robotic joints are equipped with link-sided torque sensors, which reduce the available design volume, lower the joint stiffness, and require complex cable routing. This paper presents an experimental study of a novel sensorized strain wave gear named RT1-T, which was developed by Schaeffler Technologies. The study was implemented on a joint testbed, including a high-resolution reference torque sensor at the link side. In addition to the measurement accuracy and linearity, a torque ripple analysis is performed. The joint torque control capabilities are determined along dynamic trajectories and compared to the performance achieved with a link-sided reference sensor. The sensor employed in the testbed has a static torque error of 0.42 Nm and an average closed-loop torque control error of 0.65 Nm above the reference sensor.

## 1 Introduction

Joint torque control for manipulators is a standard methodology applied in robotics, which relies on the feedback of the acting torque in the joint measured by a sensor. For joints with low-reduction gears, the acting torque could be estimated by sensing the motor current, which is commonly done in so-called quasi-direct drive actuation systems (Wensing et al., 2017). For high-reduction gears such as strain wave gears, the friction present in the gear prevents this possibility, and the applied torque needs to be measured by a link-sided torque sensor. Harmonic drive gears are a typical example of strain wave gears used in robotics, e.g., in the DLR lightweight robot (Albu-Schaeffer et al., 2007a).

The idea of sensorizing strain wave gears was already proposed in the 1980s. The advantages of using such a sensorized gear are a more compact design volume, no reduction in joint stiffness, and simplified cable routing. However, sensing the torque directly in a strain wave gear features several challenges that prevented this technology from advancing into a product.

A periodic transmission error called torque ripple is a characteristic of strain wave gears; i.e., a periodic torque is generated at the output shaft due to speed variations, even if the rotational speed of the gear’s input shaft is constant. An additional effect of sensorized strain wave gears is a torque sensing ripple resulting from unwanted strain exposure to strain gages, which results from positioning inaccuracies on the flex spline. The torque ripple is an actual applied torque to the output shaft of the gear, while the sensing ripple is only a torque measurement error (Godler et al., 2001). Reducing the sensing ripple to an acceptable level is a major challenge in development of sensorized strain wave gears.

**FIGURE 1:**
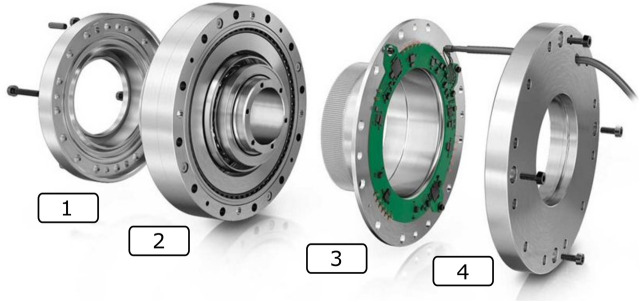
Extruded overview of the RT1-T. 1) Link-side bearing, 2) strain wave gear with a wave generator on the input-(motor-)side and circular spline on the output-(link-)side, 3) flex spline equipped with strain gages, and 4) motor-side flange toward the motor housing (Schaeffler Technologies, 2023b).

This paper introduces the novel sensorized strain wave gear RT1-T (Schaeffler Technologies, 2023b) developed by Schaeffler^1^ to the robotics community, which is depicted in Figure 1. In particular, this paper experimentally identifies the torque sensing characteristics of RT1-T in a robotic joint testbed. Sequentially, the torque signal provided by RT1-T is used in a joint torque feedback controller for static and dynamics experiments, and the control error is compared to the performance of a reference torque sensor.

The rest of the paper is organized as follows. Section 2 provides an overview of the state of the art for sensorized strain wave gears proposed to be used in robotic applications. Section 3 then reports on the mechatronic design of the RT1-T strain wave gear and specifies how the sensor signal is used in the joint torque control approach. Finally, Section 4 presents the experimental study that characterizes the linearity, accuracy, and sensing ripple of RT1-T. Moreover, friction losses in the gear are quantified, which is essential to understand the achieved sensor accuracy, and finally, the performance of the joint torque controller is presented.

## 2 State of the art: sensorized strain wave gears in robotics

The standard approach for torque sensing in robotic joints is to measure the strain in elastic elements subject to deformation by attaching strain gages. The measured voltages can be related to the torque acting on the element. Conventional joint torque sensors feature mechanical structures where the deformation can be precisely related to the acting torque. Based on this technology, various commercial products are available nowadays from multiple companies, e.g., (Sensodrive, 2023).

Hashimoto et al. (1993) exploited the flexibility of a strain wave gear to measure the acting torque by attaching strain gages on the flex spline. Despite using four pairs of strain gages, measuring the joint torque based on the flex spline deformation usually suffers from sensing ripple due to positioning errors in the strain gage placement. Moreover, complicated cable routing prevents free rotation without an end stop. The authors extended their work in Hashimoto et al. (2000) by using eight pairs of strain gages to reduce the overall sensing ripple to 2% in the torque signal of the gear with a capacity of 98 Nm even under high velocity.

Taghirad and Belanger (1998) demonstrated that the sensing ripple in sensorized strain wave gears could be substantially reduced by improving the positioning accuracy of the strain gages by using a microscope and transparent film. The authors also addressed the problem of torque ripple reduction using a Kalman filter; however, the proposed method introduces a time delay and cannot distinguish between the torque ripple induced by the gear meshing vibrations and the sensing ripple in the measurement signal resulting from the measurement principle itself.

Godler et al. (2001), Godler and Hashimoto (1998), and Godler et al. (2000) showed that an odd number of strain gages achieve better results in sensing ripple compensation, while requiring less positioning accuracy for the strain gages. They proposed a gain tuning method for the voltages of different strain gages to compensate for the sensing ripple. A torque-sensing error of approximately ± 1.5 Nm was demonstrated using a strain wave gear with an instantaneous torque capacity of 98 Nm.

Sensinger and Weir (2006) proposed a combination of classical Wheatstone bridge configurations for the strain gages in combination with gain tuning approaches to reduce the sensing ripple. The experimental validation showed a remaining sensing ripple of approximately ± 0.5 Nm for a strain wave gear with a rated torque of 28 Nm.

Jung et al. (2017) presented a sensorized strain wave gear to measure external torques acting on the joint. They exploit the characteristic of strain wave gears that the torque ripple frequency constitutes out of multiples of the gear input shaft velocity. An order tracking filter, in combination with a notch filter, was used to compensate for the sensing ripple. The experimental results showed a remaining maximum sensing ripple error of 4.8 Nm on a desired torque trajectory of approximately −20 Nm to 20 Nm.

Table 1 provides a quantitative summary of the achieved torque estimation performances in the reviewed state of the art; the torque errors are related to the respective applied torque range.

**TABLE 1:** Overview of the achieved torque estimation performance in the reviewed state of the art.

Reference	Applied torque range [Nm]	Torque error [%]
Hashimoto et al. (2000)	± 98	2.0
Taghirad and Belanger (1998)	± 40	5.0
Godler et al. (2001)	± 98	1.5
Sensinger and Weir (2006)	± 28	1.8
Jung et al. (2017)	± 20	24.0
RT1-T (Schaeffler Technologies, 2023c) (identified within the scope of this work)	± 55	0.8
RT1-T (Schaeffler Technologies, 2023c) (according to the datasheet)	± 143	3.0^*^

* The provided value includes cross loads and further joint-level influences.

## 3 Mechatronic setup and control

This section first provides an overview of the mechatronic design of the sensorized strain wave gear RT1-T. Subsequently, the integration of RT1-T onto the joint testbed, the testbed’s structure, and the RT1-T unit’s connection to the evaluation electronics are described. Finally, the structure of the joint torque controller for the experimental study is presented.

### 3.1 Mechatronic structure of RT1-T

**FIGURE 2:**
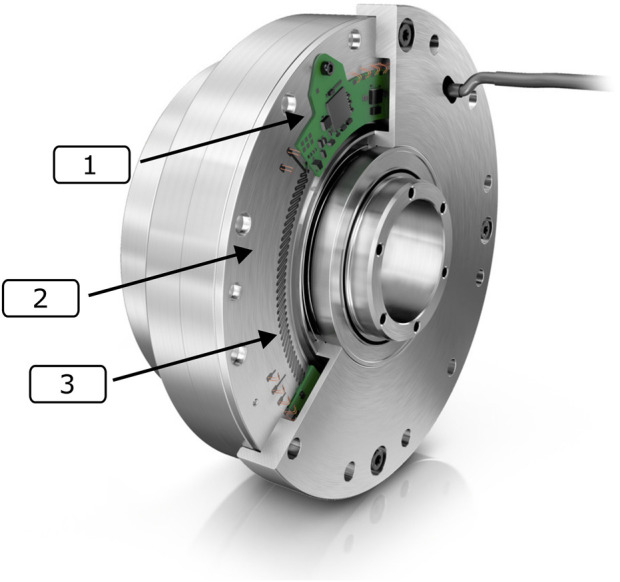
An integrated overview of the RT1-T. 1) Integrated electronic control and signal processing system, 2) flex spline, and 3) concentric circles of strain gages directly applied on the flange of the flex spline (Schaeffler Technologies, 2023c).

An extruded view of RT1-T is presented in Figure 1, whereas Figure 2 depicts specifically the location of the strain gages and their integrated electronics in the strain wave gear. In contrast to the conventional setup, which possesses a fixed circular spline, the rotating components of RT1-T are the wave generator on the input shaft toward the motor and the circular spline on the output shaft toward the joint. Therefore, the static component of RT1-T is a fixed flex spline sensor body equipped with strain gages. As the flex spline is mounted statically, the cable routing is simplified in this setup.

In operation, the joint torque is estimated by measuring the deformation of the flex spline using the strain gages that are applied directly to the metallic surface. Their application shape is several concentric circles as full bridges on the flange section of the flex spline. Due to the different pitch circle radii, the respective strain gages are exposed to different degrees of shear stress. The application is performed using the Schaeffler thin film sensor coating technology Sensotect (Schaeffler Technologies, 2023a). This technology foresees the application of the coating material to the flex spline’s entire collar sleeve, followed by a structuring of the strain gages via laser ablation. By locally removing the material, this process enables an arbitrary shape for the strain gages. It eliminates the need for subsequent trimming of the strain gage bridges via additional trim structures. As reported in Section 2, precise placement of the strain gages onto the flex spline is required to reduce torque ripple in the measurement signal. In the cited works, the placement of the strain gages was glued, which easily resulted in inhomogeneities. For example, pressing on the adhesive creates local tensions that influence the measurement signal of the strain gages. For the manufacturing of the sensorized flex spline, the coating with a layer thickness of 10 μm is applied by a sputter physical vapor deposition (PVD) process, and the strain gages are formed by laser structuring (Schaeffler Technologies, 2023c). Consequently, adhesives or transfer polymers are not required, and the resulting strain gages feature just small deviations in hysteresis, linearity, and temperature dependency.

The electronic control and signal processing PCB is directly attached to the flex spline, as visible in Figure 2. The sampling and signal processing rate, as well as the resulting torque estimate, is 5 kHz. The strain gage signals are evaluated by a neural network, which is pre-trained and afterward adapted for each sensor by transfer learning. The neural network is trained based on measurements of a link-side reference sensor for static and dynamic scenarios. Afterward, the trained neural network is adjusted for each specific device to account for manufacturing tolerances. Note that the torque estimate is computed purely on the strain gage signals without the need for position or velocity information of the flex spline state.

Finding a pure model-based approach and the corresponding parameters that map the strain gage signals to an accurate torque estimate is highly complex, as evaluated in Hashimoto et al. (1993). In order to cover the entire complexity of all different strain states, a neural network is designed to evaluate the strain gage signals. Zero velocity crossing scenarios, load changes, or torque ripple compensation are especially challenging to model. Moreover, a model-based torque computation running in parallel checks the neural network outputs for plausibility.

### 3.2 RT1-T joint testbed integration

**FIGURE 3:**
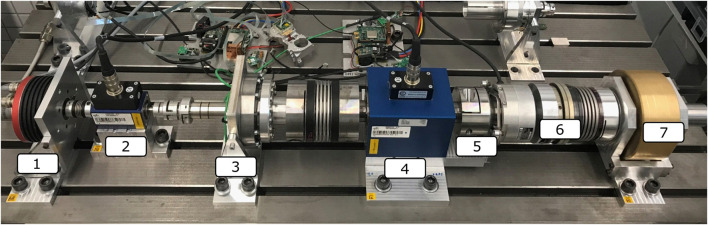
Testbed overview with 1) ILM85 motor, 2) torque sensor 5 Nm, 3) sensorized strain wave gear RT1-T, 4) torque sensor 200 Nm, 5) position encoder, 6) overload clutch, and 7) link inertia.

For the experimental study in this work, an RT1-T of type RT1-H-17-100-UHS-T is utilized with the specification provided in Table 2. On the input side, an ILM85 permanent synchronous motor from TQ-RoboDrive is used as the drive unit on the testbed (cf. Figure 3), which is controlled by an Elmo Gold Whistle Servo Drive (Elmo Motion Control, 2023). Furthermore, an incremental encoder from Heidenhain with 2048 increments/revolution and a torque sensor DR-2643 from Lorenz Messtechnik with a nominal torque of 5 Nm are installed on the drive side. The incremental encoder is placed directly on the motor, and the torque sensor is located between the motor and the gearbox, see Figure 3 (1) and (2). Subsequently, the RT1-T gear (3) is attached to the drivetrain. On the link side, a Lorenz Messtechnik DR-2643 torque sensor (4) with a nominal torque of 200 Nm is used. Both torque sensors on the input and output sides have an accuracy class of ±0.1 % of the reading end value. A position encoder from Heidenhain (5) with a resolution of 25 bits and a system accuracy of 97 μ rad is located after the torque sensor on the output side. Finally, a safety clutch (6) with a maximum release torque of 200 Nm interconnects the testbed’s output side with the link inertia. This configuration is used for the dynamic measurements to obtain the torque ripple. An additional link-sided lever arm is mounted for the torque control evaluation.

**TABLE 2:** Specification of the RT1-H-17-100-UHS-T.

Property	Value
Gear size	17
Maximum torque $\left(T_{R}\right)$ (main measurement range)	± 70 Nm
Collision torque $\left(T_{M}\right)$ (maximum measurement range)	± 143 Nm
Accuracy^*^	± 3% $T_{M}$
Resolution	16 bit

* The provided accuracy includes cross loads and further joint-level influences.

For static measurements, either the input or the output side is clamped. If the input side is fixed, the motor is transferred to the output side, and an additional planetary gear with a ratio of 1:64 connects the motor and the testbed after the Heidenhain position encoder at (5). In order to read the measurement data from the RT1-T, DLR’s evaluation electronics for digital sensors is adapted. A generic SPI Master on the FPGA of the evaluation electronic is adapted and integrated into the SpaceWire backbone bus of the testbed with a 3-kHz communication protocol cycle. MATLAB/Simulink is used for measurement recording, real-time monitoring of the actuators and sensors, and for the development and execution of the control algorithm. The Simulink model runs as a compiled C code on a real-time computer using Simulink’s Real Time Control Toolbox. The communication between motor and sensor electronics and the automatically generated executable model from Simulink is performed via an EtherCAT. The measured values of the analog torque sensors are digitized with a PCI card from National Instruments and transmitted to the real-time model. The model is controlled at runtime on a remote computer that is connected to the real-time computer. The sampling frequency of the model is 3 kHz.

### 3.3 Control

This section summarizes the joint control approach of DLR lightweight robots, which will be used in the experimental study, and was originally presented by Albu-Schaeffer et al. (2007b). The joints are modeled via a flexible model due to their low joint stiffness, e.g., induced by the strain wave gears or smaller shafts. Based on Spong (1987), the joint is modeled by two inertias interconnected by a spring-damper system as

$$\begin{array}{cc} m\ddot{q}+g & =\tau +dk^{-1}\dot{\tau }+\tau_{ext}\\ b\ddot{\theta }+\tau +dk^{-1}\dot{\tau } & =\tau_{m}-\tau_{f}\\ \tau & =k\left(\theta -q\right) \end{array},$$ (1)

where m∈R and b∈R are the inertia of the link and motor, respectively. The spring-damper system connects the rotor and link and is represented by the spring of stiffness k∈R and a damper with constant d∈R . The link and motor positions are denoted by q∈R and θ∈R , respectively. Moreover, the motor torque, spring torque, and external torque are denoted by $\tau_{m}\in R$ , τ∈R , and $\tau_{ext}\in R$ , respectively. The friction acting within the joint is summarized in $\tau_{f}\in R$ .

For joint torque control, a PD-type torque feedback approach is considered, which regulates the torque error $\tilde{\tau }\in R$ between measured τ desired torque $\tau_{d}\in R$

$$\tau_{m}=\tau_{d}-K_{T}\tilde{\tau }-K_{S}\dot{\tau },$$ (2)

with $K_{T}\in R^{+}$ and $K_{S}\in R^{+}$ being positive gain values. The closed-loop system is therefore given by substituting Eqs 1, 2.

$$b\ddot{\theta }=\left(1+K_{T}\right)\tilde{\tau }-\left(K_{S}+dk^{-1}\right)\dot{\tau }-\tau_{f}.$$ (3)

Two controller variants of Eq. 2 are implemented on the testbed. One controller uses the torque signal originating from the RT1-T within the feedback and the other uses the torque signal originating from the reference sensor.

## 4 Experimental study

Several configurations of the testbed are prepared to evaluate the performance of the RT1-T torque measurement. First, the static and dynamic characteristics of the sensor are evaluated. Afterward, the performance in a closed-loop torque controller is investigated. Finally, an application study is presented, relating the torque measurement accuracy of the RT1-T to the Cartesian force accuracy of a conventional cobot.

### 4.1 Linearity

**FIGURE 4:**
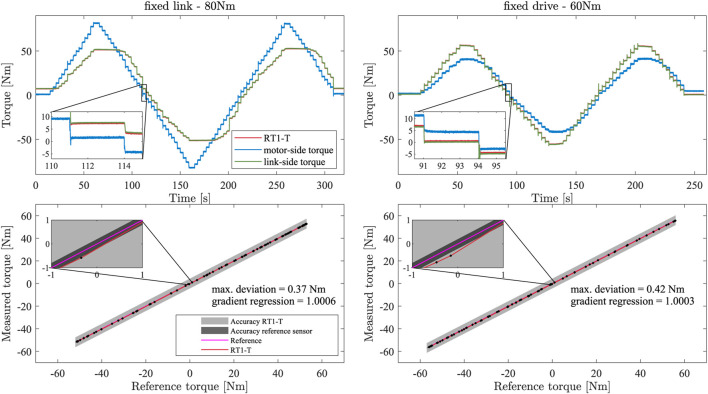
Torque measurements for fixed link side (left) and fixed motor side (right). The upper row shows the torque measurements of the RT1-T, motor-side, and link-side sensors for an applied step signal. The lower row displays the linearity and absolute deviation properties of the RT1-T for the respective scenario, including the accuracy based on the datasheet for the RT1-T and reference sensor.

The linearity of the RT1-T sensor with respect to the reference sensor is evaluated in a static scenario. First, the link side of the testbed is rotationally fixed, and the commanded motor torque is a step signal with a step height of 5 Nm and a step duration of 3 s until ± 80 Nm is reached. Afterward, the same experiment is repeated with a rotationally fixed motor side and a torque applied from the link side until ± 55 Nm is reached. Here, the lower torque magnitude results from the limitations of the used planetary gear. The measured signals of the RT1-T sensor, the motor, and the link-side reference sensors are displayed in the upper row of Figure 4. All quantities are transformed to the link side. Friction losses, mainly within the gear, lead to a lower torque measured compared with the commanded step height. For the following analyses, averaged measurement points are computed for each torque step such that transient effects can be neglected.

To evaluate the linearity, the RT1-T torque measurement is plotted against the link-side reference sensor, as shown in the lower row of Figure 4. The red curve represents the regression line fitted in the experimental data, while the pink line indicates the reference with an ideal slope of 1. The regression line features a slope of 1.0006 for the setup with the fixed link side and 1.0003 for the fixed motor side, proving a good linearity behavior within the evaluated measurement range. The maximum identified deviation from the reference torque signal is 0.37 Nm and 0.42 Nm.

### 4.2 Torque ripple analysis

A common drawback of sensorized strain wave gears is periodic error signals in the torque measurements. These error signals are referred to as ripple; the deformation of the flex spline induces them while the gear rotates. The ripple frequency is proportional to the gear input shaft rotational velocity and can be described as a periodic function of the input shaft angular position. The fundamental frequency component is twice the gear input velocity (Godler et al., 2001). Next to the torque ripple resulting from the gear meshing vibration, a fraction of the torque ripple in the torque measurement, denoted as sensing ripple, results from the measurement principle itself. The strain gages attached to the flex spline are exposed to unwanted strain resulting from the elliptical shape of the flex spline (Taghirad and Belanger, 1998). Hashimoto et al. (1991) demonstrated that at least two pairs of strain gages are required to compensate for the sensing ripple. However, this compensation method requires high positioning accuracy of the strain gages (Taghirad and Belanger, 1998).

**FIGURE 5:**
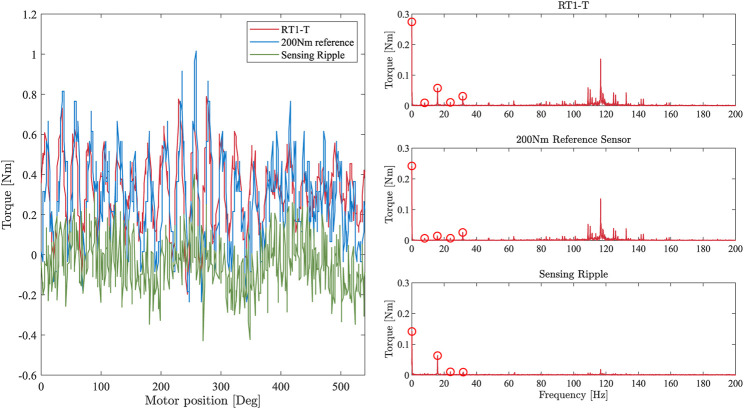
Left: torque measurements of the RT1-T and reference sensor for a constant motor velocity of 50 rad/s. The green line is the difference of both signals, indicating the sensing ripple of the RT1-T. Right: Fourier analysis applied to the torque measurements of the RT1-T and reference sensor for a constant motor velocity of 50 rad/s. The first peak corresponds to a constant signal offset; further peaks are multiples of the motor velocity.

To evaluate the sensing ripple of the RT1-T, an inertial disk (cf. Figure 3) is attached to the link side, and a constant desired motor velocity is commanded. The torque measurements of the RT1-T and the reference sensor are recorded, and a fast Fourier analysis is performed on the signals to obtain the amplitudes and phases of the fundamental frequency components. This experiment is conducted with an input shaft rotational velocity of 50 rad/s. The left side of Figure 5 shows the recorded signals of the RT1-T and the link-side reference sensor, as well as the difference between both signals. The latter contains information about possible friction losses between the two locations of torque measurement within the drivetrain, as well as measurement errors of the RT1-T signal.

The Fourier analyses of all three signals are displayed on the right side of Figure 5. As expected for strain wave gears, components with a frequency of two and four periods per input shaft revolution dominate the torque measurements of the RT1-T and the reference sensor. The Fourier analysis of the difference signal also reveals a minor sensing ripple in the torque measurement of the RT1-T sensor, which features an additional component with a frequency of two periods per input shaft revolution, i.e., 15.92 Hz and an amplitude of 0.063 Nm. Moreover, components at three and four periods per input shaft revolution, 23.89 Hz and 31.85 Hz, are identified with an amplitude of 0.01 Nm each. However, the accumulated amplitude of 0.083 Nm of this experiment, which corresponds to 0.12% of the maximum torque, has only minor effects on the torque measurement quality.

### 4.3 Friction

**FIGURE 6:**
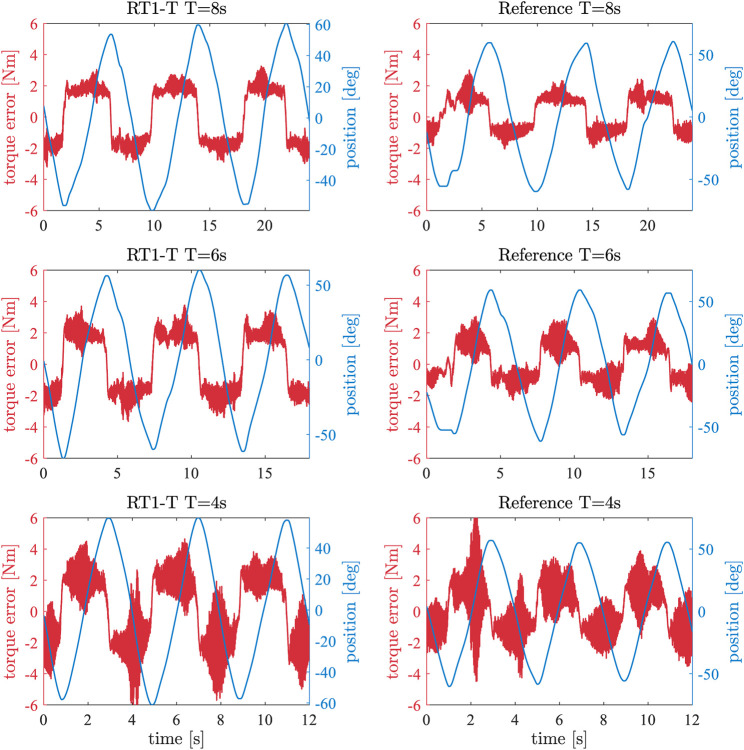
Red: closed-loop control error for applied sinusoidal position signal and $\tau_{d}=0$ Nm. Blue: sinusoidal position signal with an amplitude of ± 60 deg, applied to the link side with different period durations. The left column shows the control error for the RT1-T for different period durations, and the right column shows the one of the reference sensor, respectively.

In order to analyze the friction losses of RT1-T, a further experiment is conducted. Next to the inertial disk, an additional lever is attached to the link side of the drivetrain. Moreover, the joint is operated in torque control mode based on Eq. 2. The desired torque is set to zero, i.e., $\tau_{d}=0$ Nm, yielding that the output side can ideally be rotated with little resistance. Subsequently, sinusoidal position curves with a period of T=4 s and an amplitude of about ±60 deg are manually applied via the attached lever arm on the output side. Figure 6 shows the position profile and the torque measurements of the RT1-T and reference sensor over time, respectively.

**FIGURE 7:**
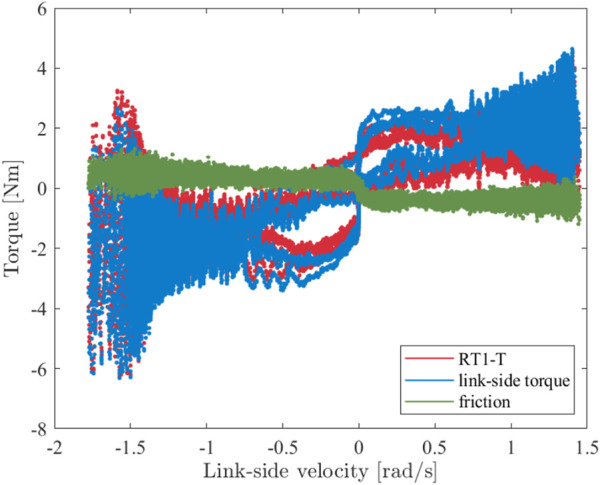
Torque measurements of RT1-T and reference sensor for manually applied sinusoidal oscillation with T=4 s and an amplitude of ± 60 deg. The difference between both signals is displayed in green and indicates the friction losses between the two measurement locations.

Due to the specific design of the RT1-T sensor, the friction of the output shaft bearing cannot be measured by the strain gages attached to the flex spline. To identify the friction losses, the measured torque signals are plotted against the motor velocity, as shown in Figure 7. The difference between both signals is depicted in green and represents the friction in the bearing. Coulomb and viscous friction effects can be directly identified; further components, such as torque-dependent effects, were not investigated. The following static friction model is fitted into the recorded data:

$${\hat{\tau }}_{f}=\tau_{f,c}+\alpha_{v}\dot{\theta },$$ (4)

where ${\hat{\tau }}_{f}$ is the estimated friction loss, $\tau_{f,c}$ represents the Coulomb’s friction, and $\tau_{f,v}=\alpha_{v}\dot{\theta }$ the velocity-dependent viscous friction. A least-squares optimization applied to the friction model in Eq. 4, results in $\tau_{f,c}=0.24$ Nm and $\alpha_{v}=0.17$ Nms/rad. It can be observed that the correlation between the torque measurements of the RT1-T and the reference sensor is also maintained for higher velocities despite increasing dynamical effects in the drivetrain; see Figure 7. The RT1-T does not suffer from a pronounced internal dynamic influencing the torque estimation for the evaluated frequency range.

### 4.4 Torque control performance

In order to analyze the performance of the RT1-T sensor within a closed-loop controller, the experiment from Section 4.3 is executed for different periods (T ∈{10, 8, 6, 4, 3, 2, 1.5} s). Here, sinusoidal position profiles are applied to the link side twice, once with the torque control loop closed with the RT1-T sensor and once with the reference sensor. The controller parameters are given in Table 3.

**TABLE 3:** Parameters of the control law in Eq. 2 for using the RT1-T or the reference sensor for torque measurement.

Parameter	RT1-T	Reference sensor
$K_{T}$ [ ]	9	12
$K_{S}$ [s]	0.03	0.03

**FIGURE 8:**
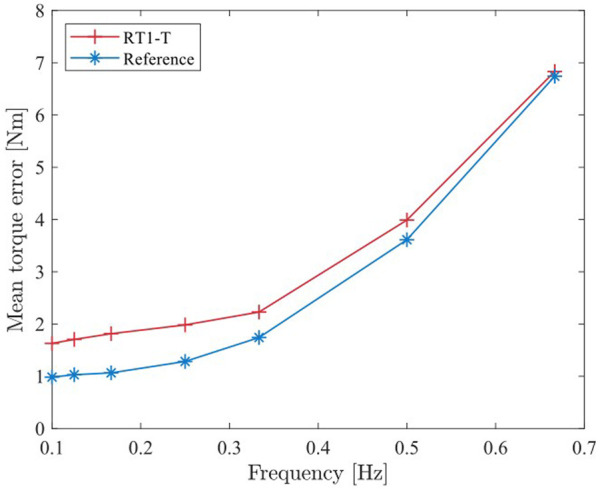
Mean closed-loop control error of the RT1-T and the reference sensor for different periods based on the torque measurements of Figure 6.

Figure 6 shows a subset of the conducted iterations. Note that this experiment has only qualitative character since the acceleration profiles do not match precisely for the RT1-T and reference sensor iterations. For each iteration, the absolute control error is computed, i.e., the deviation of the reference sensor torque measurement to the desired torque ( $\tau_{d}=0$ Nm). In order to achieve better comparability, the mean torque error for each iteration is computed and plotted against the corresponding frequency of the lever arm motion; see Figure 8. The mean torque error when using the RT1-T sensor is, on average, 0.65 Nm higher than when using the reference sensor for frequencies up to 0.35 Hz. Next to the sensing ripple in the RT1-T sensor, the friction of the bearing at the output shaft of the gearbox influences the control performance. In addition, the inertial effects of the mechanical elements between the different locations of torque measurements should be considered. For both sensors, the control errors increase above a frequency of 0.35 Hz due to a saturation of the motor torque.

### 4.5 Application study

**FIGURE 9:**
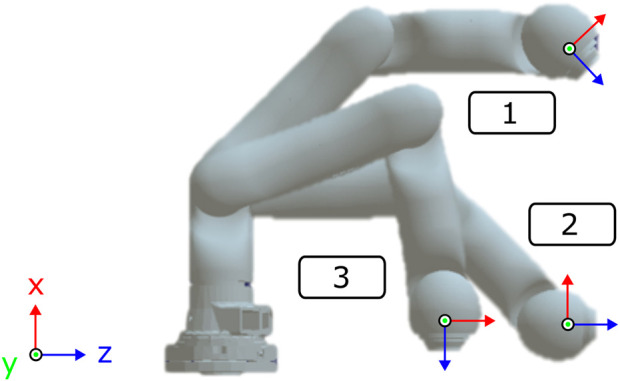
Simulation of different joint configurations with the KUKA LWR IV+ with seven torque-controlled joints.

In this example, we relate the torque measurement accuracy of the RT1-T to a resulting Cartesian tool center point (TCP) force accuracy for a cobot with seven degrees of freedom. We utilize the kinematic structure of the commercially available KUKA LWR IV+; see Figure 9. Table 4 provides the results for different joint configurations. We assume a torque measurement accuracy of ± 0.4 Nm in every joint and always consider the worst-case scenario for each Cartesian axis; i.e., depending on the sign of the respective Jacobian entry, we choose a torque measurement accuracy of +0.4 Nm or −0.4 Nm. The maximum Cartesian TCP force accuracy is configuration-dependent and 4.59 N for configuration 1. A cobot equipped with RT1-T units features a mean Cartesian TCP force accuracy of 2.65 N in the tested configurations. In addition, the relative Cartesian TCP force accuracy is provided, relating the maximum Cartesian TCP force accuracy to the maximum measurement range of 143 Nm per joint.

**TABLE 4:** Worst-case static TCP force accuracy in absolute and relative values for different joint configurations assuming a torque accuracy of 0.4 Nm per joint. The relative force accuracy is computed with respect to the maximum measurement range of 143 Nm per joint mapped to the TCP. Here, the kinematics of the KUKA LWR IV+ is used.

Configuration [deg]	Cartesian TCP force accuracy [N]	Cartesian TCP relative force accuracy [%]
$q_{1}=\left(0,\;-45,\;0,\;45,\;0,\;-45,\;0\right)$	$f_{1}=\left(4.59,\;1.31,\;2.10\right)$	(1.28,0.37,0.59)
$q_{2}=\left(0,\;-90,\;0,\;45,\;0,\;45,\;0\right)$	$f_{2}=\left(1.51,\;3.76,\;4.30\right)$	(0.42,1.05,1.20)
$q_{3}=\left(0,\;-70,\;0,\;100,\;0,\;-10,\;0\right)$	$f_{3}=\left(2.28,\;2.06,\;1.97\right)$	(0.64,0.58,0.55)

## 5 Discussion and future work

This paper presented an experimental study that characterized a novel sensorized strain wave gear termed RT1-T developed by Schaeffler and investigated its performance for joint torque control in robotics applications. The presentation of the state-of-the-art sensorized strain wave gears emphasized that prior work on sensorized strain wave gear exists; however, it has yet to result in a product suitable for robotics application, e.g., due to applied manufacturing techniques and their associated impact on the achieved inadequate accuracy. The RT1-T overcomes the shortcomings mentioned above by an industrially controlled process that enables precise sputtering of the strain gages used to sensorize the flex spline of the strain wave gear. A neural network uses the signals to estimate the transmitted torque of the gear.

The characterization of RT1-H-17-100-UHS-T is subsequently performed in a joint-level testbed, allowing for a comparison with a high-accuracy link-sided torque sensor. Static experiments performed in two configurations, “fixed link side” and “fixed drive side,” confirmed sensor accuracy between 0.37 Nm and 0.42 Nm, and a linearity deviation between 0.03% and 0.06% with respect to the reference sensor. In dynamic measurements using a link-sided inertia, the sensing ripple, which is purely dependent on input position, was found to be 0.083 Nm and independent of the input speed. Within the calibration phase of RT1-T, the parameters of a neural network are trained to estimate the link-sided torque. In the measurements of this work, a velocity-dependent offset in the torque estimate, e.g., due to not accounted frictional effects, remains and could be approximated by a friction model with 0.24 Nm Coulomb’s friction and 0.17 Nms/rad viscous friction.

The joint torque control performance is compared using the RT1-T and the reference torque sensor in a feedback loop. Using periodic trajectories with frequencies ranging up to 0.7 Hz, the torque control error is established to 0.65 Nm on average.

In conclusion, the RT1-T has an absolute torque measurement error of 0.42 Nm ^2^ in static experiments and an average torque control error of 0.65 Nm, in each case related to the reference sensor of the testbed. Based on these values and the underlying experiments, the sensor is evaluated as suitable for use in a lightweight robot.

### 5.1 Potential application cases

Sensorized strain wave gears with torque-sensing characteristics comparable to the RT1-T, are suitable alternatives to state-of-the-art link-side torque sensors as they do not introduce additional elasticity into the robotic joint and could increase the control performance. Moreover, they require fewer components and less design volume, which can lead to more compact and less complex designs. A drawback of sensorized strain wave gears for joint torque feedback control is the sensor’s location with respect to the drivetrain. Effects of link-sided bearings, mechanically located after the sensor, cannot be measured and need to be estimated, which is challenging due to the nonlinear elements of friction.

The arrangement of the RT1-T type of sensorized strain wave gears, having a fixed flex spline on which the strain gages are sputtered, is advantageous since it results in a fixed sensor cable and enables infinite rotation of the link side. Applying link-sided torque sensors usually constrains the number of possible rotations since rotating sensor cables are mechanically challenging to handle.

### 5.2 Future work

In future research on this mechatronic component, two extensions are proposed which are directly related to the shortcomings of this work. The experiments have focused on establishing the characteristics of the sensorized strain gear RT1-T and the performance of its torque measurement as a feedback signal in a model-based joint torque control approach for lightweight robotic arms. A link-sided actuator is required to enable more insights into the characteristics of RT1-T under different loading conditions. This would enable the impress of different motor or link-sided load profiles to investigate to which extent the characteristics established in this work are independent of the loading condition.

To assess the joint torque control performance, corresponding experiments were conducted using a free-motion scenario. Contact scenarios, especially when the link side is in hard contact, and the corresponding controller performance should be investigated for future work. Here, the stiffer drivetrain of RT1-T compared to a drivetrain with a link-sided torque sensor could exploit its full potential.

## Data Availability

The raw data supporting the conclusions of this article will be made available by the authors, without undue reservation.

## Footnotes

1 The full name Schaeffler Technologies AG & Co. KG is omitted for compactness

2 An accuracy comparison with state-of-the-art sensorized strain wave gears is provided in Table 1.

## References

[B1] Albu-Schaeffer A. Haddadin S. Ott C. Stemmer A. Wimboeck T. Hirzinger G. (2007a). The dlr lightweight robot: design and control concepts for robots in human environments. Ind. Robot. 34, 376–385. 10.1108/01439910710774386

[B2] Albu-Schaeffer A. Ott C. Hirzinger G. (2007b). A unified passivity-based control framework for position, torque and impedance control of flexible joint robots. Int. J. Robotics Res. 26, 23–39. 10.1177/0278364907073776

[B3] Elmo Motion Control (2023). Elmo Whistle: ultra compact smart servo drive. Available at: https://www.elmomc.com/product/whistle/ (Accessed October 15, 2023).

[B4] Godler I. Hashimoto M. (1998) “Torque control of harmonic drive gears with built-in sensing,”in IECON ’98. Proceedings of the 24th Annual Conference of the IEEE Industrial Electronics Society, Aachen, Germany, August 31–September 4, 1998 (IEEE), 2, 1334–1339. 10.1109/IECON.1998.723004

[B5] Godler I. Horiuchi M. Hashimoto M. Ninomiya T. (2000). Accuracy improvement of built-in torque sensing for harmonic drives. IEEE/ASME Trans. Mechatronics 5, 360–366. 10.1109/3516.891047

[B6] Godler I. Ninomiya T. Horiuchi M. (2001). Ripple compensation for torque sensors built into harmonic drives. IEEE Trans. Instrum. Meas. 50, 117–122. 10.1109/19.903888

[B7] Hashimoto M. Ishizuka T. Godler I. Horiuchi M. (2000). “Velocity dependence of the characteristics of harmonic drive built-in torque sensing,” in Proceedings 2000 ICRA. Millennium Conference. IEEE International Conference on Robotics and Automation, San Francisco, CA, USA, April 24–28, 2000 (IEEE), 2, 1334–1339. 10.1109/robot.2000.844783

[B8] Hashimoto M. Kiyosawa Y. Hirabayashi H. Paul R. (1991). “A joint torque sensing technique for robots with harmonic drives,” in Proceedings. 1991 IEEE International Conference on Robotics and Automation, Sacramento, CA, USA, April 9–11, 1991 (IEEE), 1034–1039. 10.1109/ROBOT.1991.131728

[B9] Hashimoto M. Kiyosawa Y. Paul R. (1993). A torque sensing technique for robots with harmonic drives. IEEE Trans. Robotics Automation 9, 108–116. 10.1109/70.210802

[B10] Jung B.-J. Kim B. Koo J. C. Choi H. R. Moon H. (2017). Joint torque sensor embedded in harmonic drive using order tracking method for robotic application. IEEE/ASME Trans. Mechatronics 22, 1594–1599. 10.1109/tmech.2017.2694039

[B11] Schaeffler Technologies (2023a). Measurement of force and torque with Sensotect. Available at: https://www.schaeffler.com/remotemedien/media/_shared_media/08_media_library/01_publications/schaeffler_2/datasheet_1/downloads_4/pdb_55_de_en.pdf (Accessed October 15, 2023).

[B12] Schaeffler Technologies (2023b). Precision strain wave gears RT1-T. Available at: https://www.schaeffler.com/remotemedien/media/_shared_media/08_media_library/01_publications/schaeffler_2/datasheet_1/downloads_4/pdb_67_de_en.pdf (Accessed October 15, 2023).

[B13] Schaeffler Technologies (2023c). Precision strain wave gears - Series RT. Available at: https://www.schaeffler-industrial-drives.com/en/news_media/media_library/downloadcenter-detail-page.jsp?id=87873043 (Accessed October 15, 2023).

[B14] Sensinger J. Weir R. (2006). Improved torque fidelity in harmonic drive sensors through the union of two existing strategies. IEEE/ASME Trans. Mechatronics 11, 457–461. 10.1109/tmech.2006.878540

[B15] Sensodrive (2023). Torque sensors from Sensodrive. Available at: https://www.sensodrive.de/products/torque-technology-torque-sensors.php (Accessed October 15, 2023).

[B16] Spong M. W. (1987). Modeling and control of elastic joint robots. J. Dyn. Syst. Meas. Control 109, 310–318. 10.1115/1.3143860

[B17] Taghirad H. Belanger P. (1998). Torque ripple and misalignment torque compensation for the built-in torque sensor of harmonic drive systems. IEEE Trans. Instrum. Meas. 47, 309–315. 10.1109/19.728840

[B18] Wensing P. M. Wang A. Seok S. Otten D. Lang J. Kim S. (2017). Proprioceptive actuator design in the mit cheetah: Impact mitigation and high-bandwidth physical interaction for dynamic legged robots. IEEE Trans. Robotics 33, 509–522. 10.1109/tro.2016.2640183

